# Vestibular migraine: course of symptoms during a four-year follow-up

**DOI:** 10.3389/fneur.2025.1567233

**Published:** 2025-04-10

**Authors:** Nese Celebisoy, Aysın Kısabay, Hüseyin Nezih Özdemir, Figen Gokcay, Aysegul Seyma Sarıtas, Hulya Toydemir, Vıldan Yayla, Ilksen Isıkay, İrem Erkent, Ceyla Atac, Sebnem Bıcakcı, Feray Güleç, Dılek Top Kartı, Eylem Ozaydın Goksu

**Affiliations:** ^1^Department of Neurology, Ege University, Bornova, Türkiye; ^2^Department of Neurology, Manisa Celal Bayar University, Manisa, Türkiye; ^3^Department of Neurology, Bakırköy Dr. Sadi Konuk Education and Research Hospital, University of Health Sciences Istanbul, Istanbul, Türkiye; ^4^Department of Neurology, Hacettepe University, Ankara, Türkiye; ^5^Department of Neurology, Izmir Bozyaka Training and Research Hospital, Izmir, Türkiye; ^6^Department of Neurology, Çukurova University, Adana, Türkiye; ^7^Department of Neurology, Izmir Tepecik Training and Research Hospital, Izmir, Türkiye; ^8^Department of Neurology, Izmir Atatürk Education and Research Hospital, University of Health Sciences, Izmir, Türkiye; ^9^Department of Neurology, Antalya Training and Research Hospital, Antalya, Türkiye

**Keywords:** vestibular migraine, vertigo, headache, aural symptoms, menopause, motion sickness, family history of migraine, allodynia

## Abstract

**Background and objective:**

Data about the prognosis of vestibular migraine (VM) is scarce. VM patients on follow-up for at least 4 years were included in this multicenter study to evaluate the course of symptoms.

**Methods:**

This is a cross-sectional study. A structured questionnaire was used inquiring demographic features, age of onset of migraine headaches and vertigo attacks, headache and vertigo attack frequency, severity, associated features and the presence of interictal dizziness and positional vertigo. Menopause, history of motion sickness, and family history of migraine were recorded. Answers of the first visit were compared with the answers of the last visit. In addition, variables considered were evaluated regarding their effect on the symptom course.

**Results:**

203 patients were studied. Median vertigo and headache attack frequency and severity had significantly dropped during follow-up (*p* < 0.01 for all comparisons). Complete resolution was reported by only 5.4%. Dizziness between the attacks was present in 67%, and positional vertigo was reported by 20.2%. Univariate analysis showed that aural symptoms (*p* = 0.013) and menopause (*p* = 0.016) were risk factors for ongoing frequent vertigo attacks. A history of motion sickness (*p* = 0.019) and a family history of migraine (*p* = 0.004) were associated with the risk of frequent migraine headaches. The presence of allodynia (*p* = 0.002) was associated with severe headache attacks while an early age of onset of vertigo attacks (*p* = 0.005) was a risk factor for continuing high-frequency vertigo attacks.

**Conclusion:**

Though the frequency and severity of headache and vertigo attacks decrease, complete resolution is reported by a minority.

## Introduction

Vestibular migraine (VM) defines vestibular symptoms causally related to migraine. The attacks can be associated with spontaneous or positional vertigo. Visually induced dizziness and head motion intolerance are common features (1). The diagnosis is mainly based on clinical history and the exclusion of other causes. In the absence of available and reliable diagnostic laboratory tests, the diagnosis is made using diagnostic criteria developed through the collaboration of the International Headache Society and the International Barany Society for Neuro-otology (2, 3). It is recognized as the most frequent cause of recurrent vertigo (4, 5), with a lifetime prevalence around 1% and a 1-year prevalence of 0.9% in the general population (4). In a more recent population-based survey, the prevalence of VM in adults was estimated to be 2.7% (6). Treatment strategies are based on migraine guidelines. A review of prophylactic agents reported improvement with all treatment options. However, the heterogeneity of study design and outcome reporting prohibited the establishment of a preferred treatment modality (7). There are only a few studies dealing with the prognosis of VM, and the natural course is obscure. The aim of the current study was to evaluate the course of symptoms in patients with VM.

## Materials and methods

**Figure 1:**
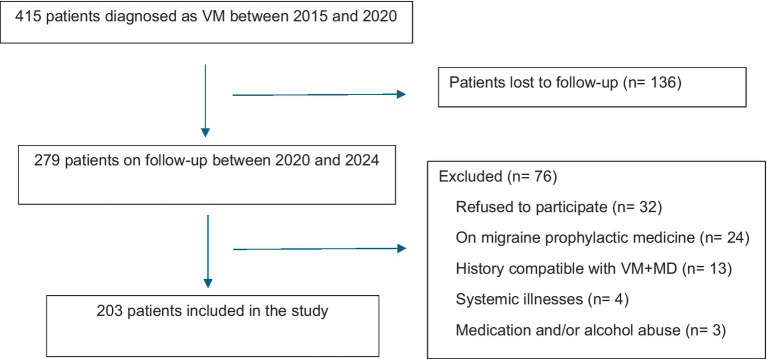
Selection of patients included in the study. VM, Vestibular migraine; MD, Meniere’s disease.

This is a cross-sectional study. The study was performed in nine tertiary centers in Turkey. Of the 415 patients who were examined and diagnosed with VM according to the criteria of the International Headache Society and the International Barany Society for Neuro-otology collaboration (2, 3), and who had participated in our previous study (8), attempts were made to reach them by phone calls. Those fulfilling the inclusion criteria were requested to answer the questions of the structured questionnaire that had been used during the initial visit. The patients were monitored during the follow-up period and received acute and prophylactic treatment when necessary. Senior neurologists evaluated the patients for inclusion in the study. The inclusion criteria were as follows: (1) definite diagnosis of VM; (2) a follow-up of at least 4 years; (3) not being on any migraine prophylactic drug during the last 3 months preceding the evaluation, after a minimum of 4 years of follow-up; (4) ability to understand and participate in the assessments. Patients with the following criteria were excluded from the study: (1) fulfilling diagnostic criteria for definite Meniere’s disease (MD) at the initial visit (9); (2) having another central or peripheral vestibular disorder (e.g., stroke, benign paroxysmal positional vertigo) within the last 3 months that may confound the results; (3) presence of severe systemic diseases (e.g., cardiovascular, diabetes mellitus with autonomic neuropathy) that may confound the results; (4) significant substance abuse or alcoholism (Figure 1).

By using the questionnaire, the course of the symptoms was evaluated. Patients were asked retrospective questions over the phone about their complaints in the last 3 months, in accordance with the inclusion criteria. Migraine type was noted as migraine with aura (MwA) or migraine without aura (MwoA). Gender, age of onset of migraine headaches and vertigo attacks, headache and vertigo attack frequency (assessed by the number of attacks per month), severity [determined by visual analog scales (VAS), measured in centimeters from 0 to 10], and associated symptoms (nausea, vomiting, photophobia/phonophobia, allodynia, tinnitus, ear fullness, hearing loss) were considered. Interictal complaints, such as dizziness and positional vertigo, were questioned. The presence of menopause, history of motion sickness, and family history of migraine were also recorded.

The outcome measures were vertigo attack frequency per month, vertigo attack severity, headache attack frequency per month, and headache attack severity. The number of attacks in the last 3 months was asked to determine the attack frequency. For VAS, the patient was asked to rate both headache and vertigo attack severity on a scale from 0 to 10. Only headaches and vestibular symptoms associated with VM were considered. Migraine-associated headaches were determined using the International Headache Society criteria (2). Vestibular symptoms associated with VM were identified based on the qualifying vestibular symptoms for VM, as outlined in the notes of the diagnostic criteria (2, 3). Attacks were defined as episodes of moderate or severe intensity symptoms lasting for designated durations, specific to either headache or vestibular symptoms (2, 3).

The answers to the questions evaluating the symptoms obtained during the first visit were compared with those gathered at the last visit to determine if any differences were present during the minimum four-year follow-up. An improvement in an outcome measure was defined as a reduction of more than 50%, in analogy with migraine treatment trials, which take a 50% decrease in migraine attacks as a success for migraine prophylaxis (10). The effect of the above-mentioned variables on improvements in the outcome measures was investigated using appropriate statistical tests.

The study protocol was approved by Ege University Medical School Ethics Committee (reference number: 24-12T/46) and approval from the local ethics committees of all the participating centers were obtained. Written informed consent was given by all participants.

### Statistical analysis

The SPSS 21 for Windows (SPSS Inc., Chicago, IL, United States) software was used for the statistical analysis. Numeric variables with a normal distribution were described using means and standard deviations, while those without a normal distribution were described using median values and ranges. The Shapiro–Wilk test was used to assess the normality of distribution. Mann–Whitney U-test was used for comparison of independent variables. Univariate regression analysis was used to assess the effect of the independent variables on the outcome measurements. Receiver operating characteristic (ROC) curve analysis was used to identify potential cut-off quantitative values determining the age to assess outcome measurements. Values of *p* < 0.05 were considered statistically significant.

## Results

**Figure 2:**
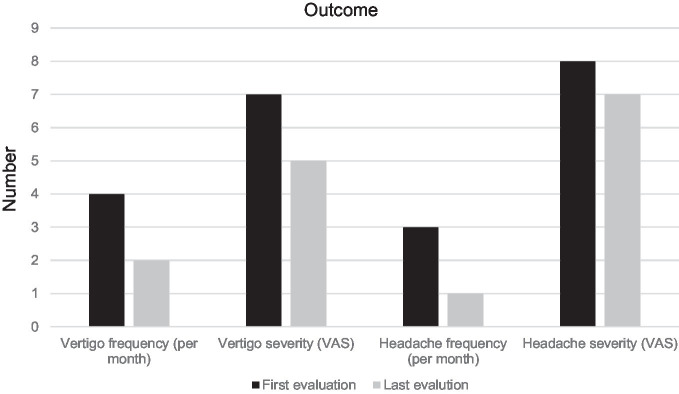
Vertigo and headache frequency and severity at the initial and last visit.

A total of 203 patients were included in the study, consisting of 173 women (85.2%) and 30 men (14.8%), with a mean age of 47 ± 12.2 years. The mean age at headache onset was 28.7 ± 6.9 years, and the mean age at the onset of vestibular attacks was 34.9 ± 9.7 years. Among the patients with migraine headaches, 171 (84.2%) had migraine without aura (MwoA), and 32 (15.8%) had migraine with aura (MwA) (Table 1). Of the 203 VM patients included in the study, 146 (71.9%) had used prophylactic medication when needed during the follow-up period and had discontinued it at least 3 months prior to the evaluation, as they no longer required it. When the symptom course was evaluated, 130 (64.1%) patients reported an improvement in vertigo attack frequency, while 73 (35.9%) patients reported no improvement. Vertigo attack VAS scores improved in 118 (58.1%) patients and did not improve in 85 (41.8%) patients (Table 2). Eleven patients (6 women and 5 men) (5.4%) in the group showing improvement defined complete resolution of both headache and vertigo attacks during the follow-up period. The median vertigo attack frequency per month was 4 (range: 1–8) at enrollment, and reduced to 2 (range: 0–8) at the follow-up assessment. The median vertigo severity was 7 (range: 1–10) according to the VAS at the onset of VM, and 5 (range: 0–10) at the follow-up. The median vertigo attack frequency and severity were significantly lower at the follow-up assessment compared to the onset (*p* < 0.001 for both comparisons) (Table 3; Figure 2). Correlation analysis showed that the decrease in VAS scores for headache and vertigo attacks were correlated (*p* = 0.03) as well as the decrease in headache and vertigo attack frequency (*p* < 0.001).

**Table 1:** Demographic and clinic features of patients.

Feature	Patients *n* = 203
Gender	
Female, *n* (%)	173 (85.2)
Male*, n* (%)	30 (14.8)
Age, years, mean ± SD	47 ± 12.2
Age at Onset of Headache, years, mean ± SD	28.7 ± 6.9
Age at Onset of Vestibular Attacks, years, mean ± SD	34.9 ± 9.7
Migraine type	
Migraine without aura, *n* (%)	171 (84.2)
Migraine with aura, *n* (%)	32 (15.8)
History of Motion Sickness, *n* (%)	124 (61.1)
Family History of Migraine, *n* (%)	57 (28.1)
Presence of menopause in women, *n* (%)	72 (41.6)
During the first evaluation, *n* (%)	37 (21.4)
Within the last 4 years, *n* (%)	35 (20.2)

*n: number VAS: visual analog scale.*

**Table 2:** Course of symptoms during follow-up.

Symptom	Patients with Improvement	Patients without Improvement
Vertigo attack frequency per month, *n* (%)	130 (64.1)	73 (35.9)
Vertigo attack severity, VAS, *n* (%)	118 (58.1)	85 (41.9)
Headache attack frequency per month, *n* (%)	132 (65.1)	71 (34.9)
Headache attack severity, VAS, *n* (%)	108 (53.2)	95 (46.8)

**Table 3:** Vertigo and headache and attack frequency and severity during the first and last evaluation.

Symptom	Evaluation at onset	Evaluation on follow-up	*P* value
Vertigo attack frequency per month, median (min- max)	4 (1–8)	2 (0–8)	< 0.001
Vertigo attack severity, VAS, median (min- max)	7 (1–10)	5 (0–10)	< 0.001
Headache attack frequency per month, median (min- max)	3 (1–7)	1 (0–7)	< 0.001
Headache attack severity, VAS, median (min- max)	8 (2–10)	7 (0–10)	< 0.001

Dizziness between attacks was present in 136 (67%) patients. Twenty-two of the 136 patients experienced new-onset interictal dizziness. Additionally, 41 (20.2%) patients reported positional vertigo, with 11 defining it as a new symptom.

An improvement in headache attack frequency was present in 132 (65.1%) patients, while 71 (34.9%) patients did not report an improvement. Headache VAS scores decreased in 108 (53.2%) patients, while 95 (46.8%) patients did not experience a decrease (Table 2). The median headache attack frequency per month was 3 (range: 1–7) at the onset of VM, and 1 (range: 0–7) at the last visit. The median headache VAS score was 8 (range: 2–10) at the onset, and 7 (range: 0–10) at the last visit (*p* < 0.001 for both comparisons) (Table 3; Figure 2).

Tinnitus and ear fullness were present in 103 (50.7%) patients. Nineteen patients with new-onset aural symptoms were included in the group that did not report an improvement in aural features. Hearing loss was reported by 22 (10.8%) patients, with 15 experiencing it during the first visit and 7 with new onset. Eighteen (8.9%) patients had allodynia at the onset of VM, while 29 (14.2%) patients reported new-onset allodynia associated with their attacks. Allodynia was not present in 156 (76.8%) patients.

The presence of motion sickness in the past medical history was noted in 124 (61.1%) patients. A positive family history of migraine was reported by 57 (28.1%) patients. Among the 173 women, 72 (41.6%) were in the menopausal period, with 37 (21.4%) in this stage at the first visit and 35 (20.2%) within the last 4 years (Table 1).

The univariate analysis revealed factors associated with vertigo attack frequency and severity. Patients with aural features (aural fullness/tinnitus, hearing loss) had a higher risk of no improvement in both vertigo attack frequency (OR: 4.500 [95% CI: 1.010–12.536], *p* = 0.013) and severity (OR: 2.438 [95% CI: 1.117–7.743], *p* = 0.032). Similarly, the presence of menopause elevated the risk of no improvement in both vertigo attack frequency (OR: 1.877 [95% CI: 1.005–3.495], *p* = 0.016) and severity (OR: 1.806 [95% CI: 1.004–3.386], *p* = 0.017).

Several factors were associated with no improvement in headache. Patients with a history of motion sickness (OR: 1.289 [95% CI: 1.028–1.615], *p* = 0.019) and a family history of migraine (OR: 1.354 [95% CI: 1.126–1.628], *p* = 0.004) had a higher risk of no improvement in headache attack frequency. The presence of allodynia (OR: 1.653 [95% CI: 1.084–2.520], *p* = 0.002) elevated the risk of no improvement in headache severity (Table 4).

**Table 4:** Results of the univariate regression analysis performed to identify factors associated with no improvement in vertigo and headache attack frequency and severity.

Outcome	Variables	Univariate Analysis
Vertigo attack frequency and severity	Aural features (aural fullness/tinnitus)	Frequency- OR: 4.500 [95% CI: 1.010–12.536]; *p* = 0.013
		Severity- OR: 2.438 [95% CI: 1.117–7.743]; *p* = 0.032
	Menopause	Frequency- OR: 1.877 [95% CI: 1.005–3.495]; *p* = 0.016
		Severity- OR: 1.806 [95% CI: 1.004–3.386]; *p* = 0.017
Headache attack frequency and severity	Motion sickness	Frequency- OR: 1.289 [95% CI: 1.028–1.615], *p* = 0.019
	Family history of migraine	Frequency- OR: 1.354 [95% CI: 1.126–1.628], *p* = 0.004
	Allodynia	Severity- OR: 1.653 [95% CI: 1.084–2.520], *p* = 0.002

Patients with and without improvement in vertigo attack frequency were compared regarding age at onset. ROC analysis was used to determine the cut-off value for age at onset. For vertigo attack frequency, an age of 39 years was determined as the cut-off value (AUC: 0.597 [95% CI: 0.526–0.665], *p* = 0.023). Patients younger than 39 years of age at the onset of VM had a higher risk of no improvement in vertigo attack frequency (OR: 1.693 [95% CI: 1.176–2.437], *p* = 0.005). No significant results were found when applying ROC analysis to vertigo severity, headache attack frequency, and headache severity (*p* > 0.05).

The other parameters considered, including gender, migraine type, associated features other than allodynia and aural symptoms, interictal dizziness, and interictal positional vertigo, were not related to an improvement in headache or vertigo attack frequency or severity (*p* > 0.05).

## Discussion

Studies dealing with prognosis in VM patients are scarce. A follow-up study conducted 9 years after the initial diagnosis found that almost 90% of the 61 patients included still suffered from recurring vestibular attacks, and migraine headaches were present in all but one patient. Vertigo frequency was reduced in 56%, increased in 29%, and unchanged in 16%. The impact of vertigo was severe in 21%, moderate in 43%, and mild in 36%. In this study, patient examinations not included in our study are also available, revealing an increase in interictal ocular motor abnormalities from 16% initially to 41% of patients at follow-up, with positional nystagmus being the most frequent finding detected in 28%. Associated cochlear symptoms had also increased from 15% initially to 49, and 18% had developed mild bilateral sensorineural hearing loss (11).

Our results were somewhat similar. Complete resolution of both headache and vertigo attacks was reported by only 5.4% of the patients. Though frequency and severity had improved, headache and vertigo attacks continued to recur. Interictal dizziness was present in 67%, and positional vertigo was reported by 20.2% of the group. Tinnitus and aural fullness were present in around 50%, with newly added patients during the follow-up period. Hearing loss was reported by 10.8%.”

In our previous study on the response to treatment in patients with VM who were given conventional migraine prophylactic drugs (amitriptyline, flunarizine, propranolol, topiramate, and venlafaxine) and followed up for at least 1 year, aural fullness and cutaneous allodynia accompanying attacks were the factors associated with reduced treatment response (12). In the current study, we did not include patients who were taking migraine prophylactic drugs to avoid interfering with the natural course of the disease. However, similar to the treatment study, the presence of aural symptoms was associated with ongoing frequent and severe vertigo attacks. VM patients are known to have a higher risk of cochlear disorders (tinnitus, sudden deafness, and sensorineural hearing loss), with a nearly three-fold increase (13), and about half of Meniere’s patients, who suffer from tinnitus, hearing loss, and aural fullness, also have a history of migraine (13). An overlap of vestibulo-cochlear symptoms in VM and Meniere’s disease (MD) has been reported in several studies (14–22). One study showed that around 50% of MD patients initially presented only cochlear symptoms without vertigo attacks (23).

The patients in our study were evaluated clinically and with pure tone audiometry during their first visit, and those with low-frequency hearing loss compatible with MD were not included in the follow-up study. Although the follow-up visits did not include an audiometric evaluation, the association of aural symptoms with more frequent and severe vestibular attacks raised suspicion for comorbid MD, which became evident over time.

Again, in another study from our group, VM patients were more frequently in the menopausal period compared to migraine patients without vestibular symptoms (24). Consistent with the above-mentioned study, menopause was a risk factor for unremitting frequent and severe vertigo attacks in the current study. A negative correlation has been shown between serum estradiol levels and the severity, frequency, and duration of VM attacks in postmenopausal women with VM (25), which aligns with our results.

In addition, an early age of onset of vestibular attacks seemed to be associated with ongoing high-frequency vertigo attacks. Central sensitization, as well as trigemino-vascular and calcitonin gene-related peptide (CGRP) effects on the vestibular system, are the theories proposed to explain VM pathogenesis (26). Significantly increased thalamic activation, in comparison with migraine patients and healthy controls, has been reported in VM patients, showing a positive correlation with the number of attacks (27). Attacks starting at an early age and continuing for years seem to be a risk factor for persistent frequent attacks.

On the other hand, a history of motion sickness and a family history of migraine were associated with ongoing frequent headache attacks. Motion sickness is well described in patients with migraine (28), and VM patients are reported to be more susceptible to motion sickness than those with migraine (24, 29, 30). The key structures in the brainstem that contribute to symptoms of motion sickness and migraine include the trigeminal nucleus caudalis, the vestibular nuclei, and the nucleus tractus solitarius. Persistent excitability of these brainstem nuclei could increase vulnerability to both motion sickness and migraine symptoms (31).

A family history of migraine is common in both migraine and VM, though not much is known about specific genetic alterations (26). A stronger link has been reported to be associated with a lower age at onset and more frequent attacks, suggesting that specific clinical features may be more determined by genetic factors (32).

The presence of allodynia in our group was associated with the risk of ongoing severe migraine headaches. Cutaneous allodynia is a common feature accompanying migraine attacks, characterized by pain perception in response to non-painful stimulation of the skin, which results from central sensitization of second and third-order trigemino-vascular neurons. It predicts a poor acute treatment response (33) and is a risk factor for disease progression from episodic to chronic migraine (34). In an online survey study including over 15,000 migraine patients, a significant association between allodynia and headache pain intensity has been reported (35).

The prognostic significance of vestibular function tests on the improvement in vertigo and headache symptoms has also been studied. Abnormal posturography and cervical vestibular-evoked myogenic potential (cVEMP) responses, indicating abnormalities in vestibulospinal pathways, have been reported to be associated with a poor response (36).

The other longitudinal studies are mainly on treatment responses with different drugs, reporting more than 70% improvement in vertigo and headache with lifestyle modifications and prophylactic medications (flunarizine, amitriptyline) (36). Female gender, comorbid benign paroxysmal positional vertigo, and high initial dizziness handicap inventory (DHI) scores were reported as good prognostic indicators for improvement in dizziness in another study with a protocol of antidepressants, antiepileptics, beta blockers, and vestibular rehabilitation (37), while peripheral vestibular weakness on caloric testing was a poor prognostic factor (38).

The main limitation of our study is that it only involves symptom evaluation, which depends on the patients’ declaration and is therefore thoroughly subjective. Additionally, the selection of patients not requiring therapy for at least 3 months, and the lack of evaluation of psychiatric disorders might be potential confounding factors. In a four-year follow-up, although the frequency and severity of the headache and vertigo attacks seemed to decrease, complete resolution was reported by only 5.4%. Interictal dizziness was reported by 67%, and positional vertigo by 20.2%. Aural symptoms and menopause were risk factors for ongoing frequent and severe vertigo attacks, while a history of motion sickness and a family history of migraine were associated with the risk of ongoing frequent migraine headaches. An early age of onset of vertigo attacks was found to be a risk factor for continuing high-frequency vertigo attacks, while the presence of allodynia was associated with severe headache attacks.

## Data Availability

The original contributions presented in the study are included in the article/supplementary material, further inquiries can be directed to the corresponding author.
